# Belladonna in Dysentery

**Published:** 1877-09-20

**Authors:** Q. C. Smith

**Affiliations:** Cloverdale, Cal.


### BELLADONNA IN DYSENTERY.

BY Q. C. SMITH, M.D., CLOVERDALE, CAL.

Deeming it unnecessary for us to speak at length of the manifold remedial virtues that have been, and are, attributed to belladonna, or to mention the numerous painful maladies which its proper administration will more or less relieve, we will endeavor to give, in a concise manner, the results of several years of our experience in its use in the treatment of dysentery.

Believing it is rarely used in the treatment of dysentery, and believing it to be the most potent agent within our reach for relieving this painful disease, we would earnestly call the attention of physicians to this special application of this valuable medicine. We have no stereotype formula by which we prescribe belladonna in dysentery, but combine it, or accompany its use, with such other remedies as may be indicated in any given case. One of our favorite combinations of it is as follows:

R. Fl. ext. belladonnæ, dr. j.
Elix. calisayæ,........ oz. iss.
Syr. ipecac.,.......... oz. ss.
M. Ft. Sol. S.—Half to one teaspoonful every one, two, or three hours, until griping pains are relieved; using at the same time, externally, a liniment made thus:

R. Fl. ext. aconite,
Chloroform,.......aa oz. ss.
Hoffman's anodyne,
Glycerine,..............aa oz. j.

M. Ft. Sol. S.—Moisten a thin cloth with the liniment and spread it over every part of the painful region; cover it *closely* with thick paper, allowing it to remain until the skin is almost blistered.

In most cases of dysentery, we commence treatment by giving small and oft-repeated doses of sulphate of magnesia, until the bowels move freely; and if the pains are severe, give the belladonna mixture, and use the liniment at the same time. After the power of the disease has been broken, should the patient be much debilitated, we frequently prescribe the following tonic mixture:

R. Elix. calisayæ,
Elix. valerinate ammoniæ, oz. j.
Tinct. nucis vomicæ,....... dr. ij.

M. Ft. Sol. S.—Half tablespoonful three or four times a day.

By way of assurance to those who are not much in the habit of using belladonna, we would say, we have never seen any permanent injury result from the slight delirium, which is more alarming than dangerous, that is often caused by its use. And its full remedial effect, in some cases, is not manifest until slight delirium or disturbance of vision is produced. A temporary cessation of the use of belladonna, for a few hours, and a moderate use of alcoholic stimulants, will relieve the delirium, after which the disease—dysentery—rarely gives much trouble.

We have treated acute dysentery according to the direction of several of the classical authorities, but the plan we have briefly outlined has been more satisfactory than any other with which we are acquainted.—*Nashville Jour. of Med. and Surg.*

